# Respiratory outcomes of metformin use in patients with type 2 diabetes and chronic obstructive pulmonary disease

**DOI:** 10.1038/s41598-020-67338-2

**Published:** 2020-06-24

**Authors:** Fu-Shun Yen, James Cheng-Chung Wei, Yu-Cih Yang, Chih-Cheng Hsu, Chii-Min Hwu

**Affiliations:** 1 Dr. Yen’s Clinic, No. 15, Shanying Road, Gueishan District, Taoyüan, 33354 Taiwan; 2 Institute of Medicine, Chung Shan Medical University, No. 110, Sec. 1, Jianguo N. Rd., South District, Taichung City, 40201 Taiwan; 3 Department of Medicine, Chung Shan Medical University Hospital, No. 110, Sec. 1, Jianguo N. Rd., South District, Taichung City, 40201 Taiwan; 4 Graduate Institute of Integrated Medicine, China Medical University, Hsueh-Shih Road, Taichung, 40402 Taiwan; 5 Management Office for Health Data, China Medical University Hospital, 3F, No. 373-2, Jianxing Road, Taichung, 40459 Taiwan; 6 College of Medicine, China Medical University, No. 110, Sec. 1, Jianguo N. Rd., South District, Taichung City, 40201 Taiwan; 7 Institute of Population Health Sciences, National Health Research Institutes, Zhunan, No. 35, Keyan Rd., Zhunan Township, Miaoli, 35053 Taiwan; 8 Department of Health Services Administration, China Medical University, No. 91, Xueshi Rd., North Dist., Taichung, 40402 Taiwan; 9 Department of Family Medicine, Min-Sheng General Hospital, No. 168, ChingKuo Rd, Taoyüan, 33044 Taiwan; 10 Section of Endocrinology and Metabolism, Department of Medicine, Taipei Veterans General Hospital, No. 201, Sec. 2 Shi-Pai Rd., Chung-Cheng Build. 11F Room522, Taipei, 11217 Taiwan

**Keywords:** Diseases, Endocrinology, Health care, Medical research

## Abstract

Few studies investigated the respiratory outcomes of metformin use in patients with coexistent type 2 diabetes mellitus (T2DM) and chronic obstructive pulmonary disease (COPD). We want to compare the long-term respiratory endpoints of metformin use and nonuse in patients with T2DM and COPD. This retrospective cohort study enrolled patients with T2DM and COPD from Taiwan’s National Health Insurance Program between January 1, 2000, and December 31, 2012. Main outcomes were hospitalized bacterial pneumonia, hospitalization for COPD, noninvasive positive pressure ventilation (NIPPV), invasive mechanical ventilation (IMV), and lung cancer. In total, 20,644 propensity score-matched metformin users and nonusers were assessed. The adjusted hazard ratios (95% confidence intervals) of metformin use relative to nonuse for bacterial pneumonia, hospitalization for COPD, NIPPV, IMV, and lung cancer were 1.17 (1.11–1.23), 1.34 (1.26–1.43), 0.99 (0.89–1.10), 1.10 (1.03–1.17), and 1.12 (0.96–1.30). Metformin use also exhibited significant dose–response relationship with respect to the risks of bacterial pneumonia, hospitalization for COPD and IMV. Consistent results were found in the sensitivity test. This nationwide cohort study demonstrated that in patients with T2DM and COPD, metformin use was associated with higher risks of pneumonia, hospitalization for COPD, and IMV. If patients with COPD use metformin, vigilance with regard to their pulmonary condition may be required.

## Introduction

Chronic obstructive pulmonary disease (COPD) is a disease of progressive inflammation in the airway with partially reversible airflow limitation^1^. Type 2 diabetes mellitus (T2DM) is characterized by insulin resistance and hyperglycemia and is considered a chronic low-grade inflammatory disease^2^. Globally, approximately 11.7% of adults and nearly 400 million people have COPD^3^; additionally, it is a leading cause of death^4^. One in 11 adults and approximately 425 million people have T2DM, making it a leading contributor to the global disease burden^5^. Approximately 1.6–16% of people with COPD have diabetes. In addition, prevalence of T2DM increases as lung function deteriorates with COPD^6^, possibly because of the inflammatory process or use of steroids in COPD treatment^7^. Nearly 10% of patients with T2DM suffer from COPD^8^. Diabetes can worsen the progression and prognosis of COPD through the consequences of hyperglycemia, including reduced respiratory function, chronic inflammation, and susceptibility to bacterial infection^9^. Effective treatment of diabetes may improve the prognosis of COPD.

Because only few studies^10^ have assessed which diabetes treatments are suitable for patients with COPD, most clinicians follow the T2DM guidelines to treat patients with COPD, with metformin typically adopted as the first-line treatment. Metformin, possibly through the activation of AMP-associated protein kinase (AMPK), can reduce the accumulation of advanced glycated end product, oxidative stress, systemic inflammation, and improve insulin resistance^11^. In addition, it can reduce airway inflammation^12^ as well as improve respiratory muscle strength^13^ and forced vital capacity^14^. One animal study revealed that metformin could reduce airway glucose permeability and limit hyperglycemia-induced bacterial growth^15^. Several studies have reported that the use of metformin in patients with T2DM and COPD can lower the risk of all-cause mortality^16–18^. Only one randomized clinical trial of metformin use studied nondiabetic patients with exacerbated COPD, and no significant difference in clinical outcomes was observed^19^; but this study was a short-term (one month) trial with moderate number (n = 52) of patients. Because the evidence regarding metformin use in patients with COPD is so inconclusive, we conducted this retrospective cohort study to evaluate the long-term respiratory outcomes of metformin use in patients with T2DM and COPD.

## Results

### Participants

**Figure 1:**
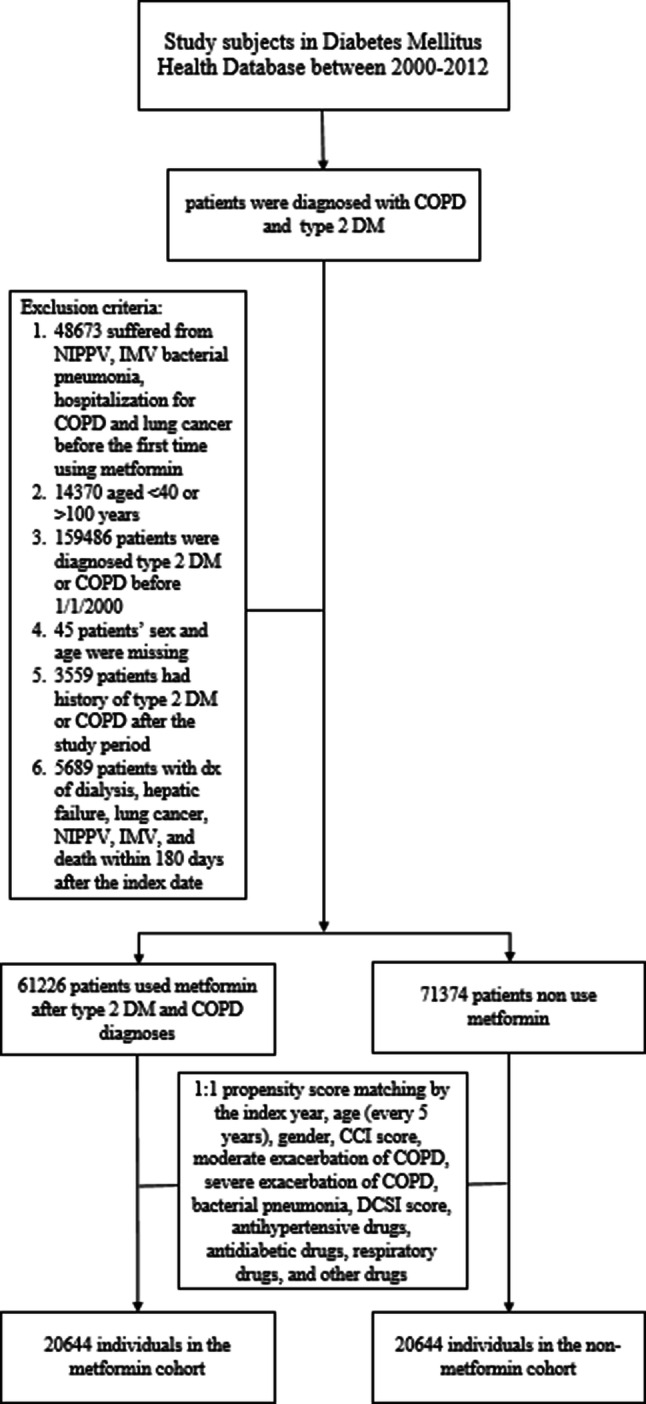
Flow chart of study design including numbers of patients.

Of the 402,153 patients in the Longitudinal Cohort of Diabetes Patients (LHDB) who were newly diagnosed with T2DM and COPD between January 1, 2000, and December 31, 2012, 61,226 and 71,374 individuals were metformin users and nonusers, respectively. These users and nonusers constituted the cohorts of our study (Fig. 1). After propensity score matching to a 1:1 ratio, 20,644 metformin users and 20,644 nonusers were included in the outcome analysis. The two groups of patients were similar with respect to all covariates (Table 1). Considered jointly, the mean age of patients in these two groups was 63.9 years. The mean follow-up times for metformin users and nonusers were 5.01 years (standard deviation [SD] = 3.26) and 5.19 years (SD = 3.11), respectively.

**Table 1:** Demographic and baseline characteristics of T2DM and COPD cohorts.

	Before propensity score matching					After propensity score matching				
	Metformin users		Metformin nonusers		Standardized difference^a^	Metformin users		Metformin nonusers		Standardized difference^a^
	(n = 71,374)		(n = 61,226)			(n = 20,644)		(n = 20644)		
	N	%	n	%		n	%	N	%	
**Gender**										
Female	32,191	45.1	27,575	45.1	0.001	9,493	45.9	9,365	45.4	0.012
Male	39,183	54.9	33,651	54.9	0.001	11,151	54.1	11,279	54.6	0.012
**Age**										
40–64 years	32,824	45.9	36,335	59.3	0.27	10,599	51.3	10,612	51.4	0.001
≥ 65 years	38,550	54.1	24,891	40.7	0.27	10,045	48.7	10,032	48.6	0.001
Mean (SD)	65.6 (12.5)		61.8 (10.7)		0.333	63.9 (11.2)		63.9 (11.2)		0
**Charlson Comorbidity Index**										
0	45,213	63.3	34,855	56.9	0.131	11,824	57.3	11,963	57.9	0.014
1	11,504	16.1	10,299	16.8	0.019	3,236	15.7	3,235	15.7	0
≥ 2	14,657	20.6	16,072	26.3	0.135	5,584	27	5,446	26.4	0.015
**Moderate exacerbations of COPD**										
0–1 in previous year	5,791	8.11	2,161	3.53	0.197	916	4.44	976	4.73	0.014
≥ 2 in previous year	65,583	91.8	59,065	96.4	0.197	19,728	95.5	19,668	95.2	0.014
**Severe exacerbations of COPD**										
0–1 in previous year	61,635	86.3	56,014	91.5	0.164	18,475	89.5	18,462	89.4	0.002
≥ 2 in previous year	9,739	13.7	5,212	8.51	0.164	2,169	10.5	2,182	10.6	0.002
**Bacterial pneumonia**										
No	51,284	71.9	52,442	85.6	0.342	17,100	82.8	16,958	82.1	0.018
Yes	20,090	28.1	8,784	14.4	0.342	3,544	17.2	3,686	17.9	0.018
**DCSI score**										
0	58,157	81.4	47,295	77.2	0.105	16,192	78.4	16,206	78.5	0.002
1	6,172	8.65	6,682	10.9	0.076	1972	9.55	1967	9.53	0.001
≥ 2	7,045	9.87	7,249	11.8	0.063	2,480	12	2,471	11.9	0.001
**Antihypertensive drugs**										
ACEI/ARB	35,440	49.6	38,415	62.7	0.266	11,242	54.4	11,318	54.8	0.007
β-Blockers	32,533	45.6	26,294	42.9	0.053	8,674	42	8,747	42.3	0.007
Calcium-channel blockers	40,170	56.2	35,542	58	0.036	11,542	55.9	11,591	56.1	0.005
Diuretics	23,479	32.9	18,724	30.6	0.05	6,075	29.4	6,068	29.4	0.001
Other antihypertensives	13,285	18.6	9,518	15.5	0.082	3,232	15.6	3,333	16.1	0.013
**Antidiabetic drugs**										
Sulfonylurea	10,342	14.5	45,923	75	1.534	8,125	39.4	8,702	42.1	0.057
Meglitinide	2002	2.8	10,395	16.9	0.489	1599	7.75	1747	8.46	0.033
DPP-4 inhibitors	1,006	1.41	11,562	18.8	0.605	817	3.96	1,060	5.13	0.033
TZDs	1,029	1.44	10,440	17	0.56	824	3.99	964	4.67	0.057
AGIs	2,468	3.46	14,139	23	0.605	1709	8.28	1899	9.2	0.026
Insulin	6,136	8.6	18,891	30.8	0.583	3,215	15.5	3,412	16.5	0.026
**Respiratory drugs**										
Short-acting β_2_ bronchodilators	3,430	4.81	2,432	3.97	0.041	830	4.02	839	4.06	0.002
Long-acting β_2_ bronchodilators	302	0.42	143	0.23	0.033	60	0.29	58	0.28	0.002
Anticholinergic agent	2073	2.9	1,153	1.88	0.067	432	2.09	444	2.15	0.004
Inhaled corticosteroids	1974	2.77	1,259	2.06	0.046	447	2.17	455	2.2	0.003
Systemic corticosteroid	22,517	31.5	14,897	24.3	0.161	4,954	24	5,005	24.2	0.006
Methylxanthine	26,573	37.2	18,565	30.3	0.146	6,211	30.1	6,259	30.3	0.005
**Other drugs**										
Statin	22,060	30.9	32,639	53.3	0.466	8,359	40.5	8,395	40.6	0.004
Aspirin	28,764	40.3	26,567	43.4	0.063	8,028	38.9	8,120	39.3	0.009

DCSI, Diabetes Complications Severity Index; ACEI, angiotensin converting enzyme inhibitor; ARB, angiotensin II receptor blocker; DPP-4, dipeptidyl peptidase-4; TZDs, thiazolidinediones; AGIs, alpha-glucosidase inhibitors.

^a^A standardized mean difference of ≤ 0.10 indicates a negligible difference between the two cohorts.

### The outcome of hospitalized bacterial pneumonia

**Figure 2:**
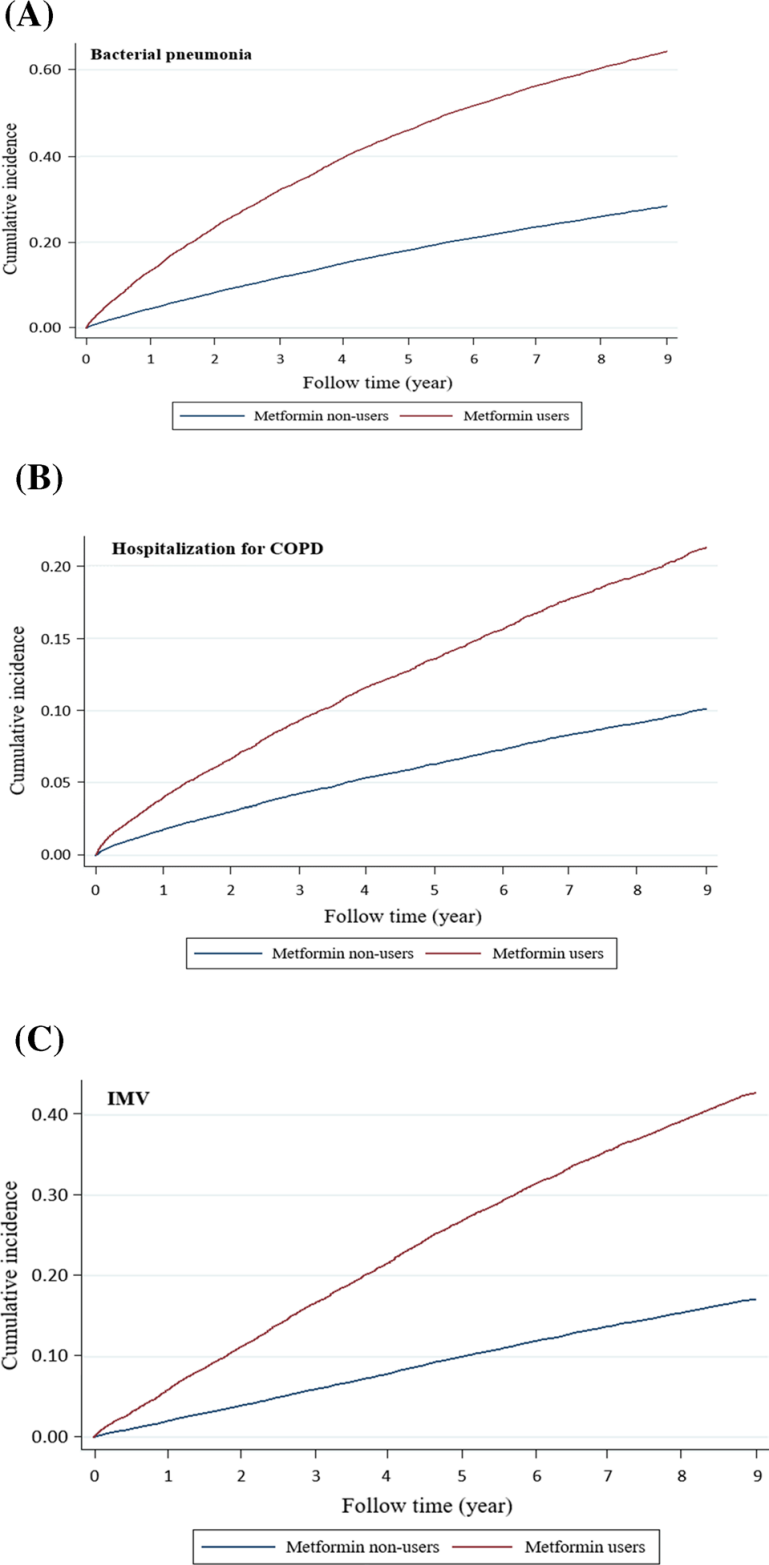
Cumulative incidence of bacterial pneumonia (**A**), hospitalization for COPD (**B**), and invasive mechanical ventilation (IMV, **C**), between metformin users and nonusers.

**Figure 3:**
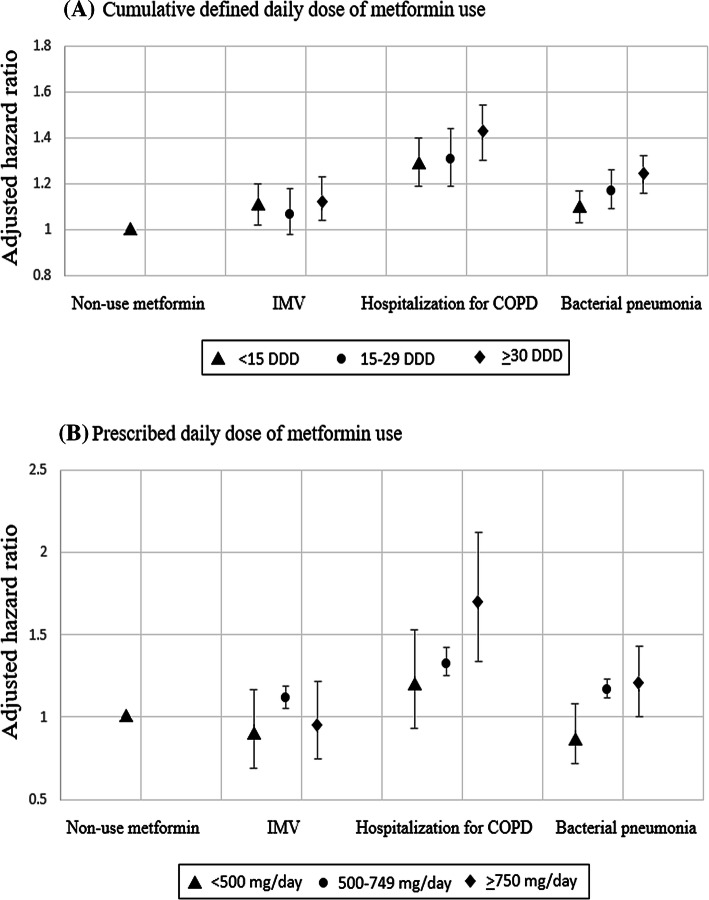
Outcomes of various dosages of metformin use relative to nonuse in patients with T2DM and COPD by (**A**) cumulative DDD and (**B**) prescribed daily dose (mg/day).

During follow-up, 3,133 (15.18%) of the 20,644 metformin users and 3,106 (15.05%) of the 20,644 metformin nonusers were admitted for bacterial pneumonia. The incidence rates (IRs) were 37.8 and 34.5 per 1,000 person-years; the adjusted hazard ratio (aHR) of metformin use vs. nonuse was 1.17, with a 95% confidence interval (CI) of 1.11–1.23 and a *p* value < 0.0001 (Table 2). The difference in the cumulative incidence of bacterial pneumonia between the metformin users and nonusers was illustrated using a Fine and Gray’s sub-distribution hazard model (Fig. 2A), which showed a higher risk of pneumonia in metformin users relative to nonusers. With respect to the risk of bacterial pneumonia, the aHRs for < 15, 15–29, and ≥ 30 cumulative defined daily dose (DDDs, relative to nonuse) were 1.09, 1.17, and 1.24, respectively (Fig. 3A); the aHRs of the prescribed daily doses of metformin, < 500, 500–749, and ≥ 750 mg/day (relative to nonuse), were 0.88, 1.17, and 1.20, respectively (Fig. 3B).

**Table 2:** Incidence rates and hazard ratios of main respiratory outcomes.

	Metformin non-users			Metformin users			Crude		Adjusted	
	Events	PY	IR	Events	PY	IR	HR (95% CI)	*p* value	HR (95% CI)	*p* value
NIPPV	710	109,526	6.48	752	106,956	7.03	1.07 (0.96–1.18)	0.18	0.99 (0.89–1.10)	0.95
IMV	1989	108,251	18.4	2,325	104,745	22.2	1.20 (1.13–1.28)	< 0.0001	1.10 (1.03–1.17)	0.001
Bacterial pneumonia	3,544	102,782	34.5	3,686	97,505	37.8	1.09 (1.04–1.14)	0.0001	1.17 (1.11–1.23)	< 0.0001
Hospitalization for COPD	2,169	105,877	20.5	2,182	101,275	21.5	1.04 (0.98–1.11)	0.12	1.34 (1.26–1.43)	< 0.0001
Lung cancer	332	109,826	3.0	392	107,488	3.6	1.20 (1.03–1.39)	0.01	1.12 (0.96–1.30)	0.12

PY, person-years; IR, incidence rate per 1,000 person-years; HR, hazard ratio; CI, confidence interval. Models adjusted by gender, age, Charlson Comorbidity Index, moderate exacerbations of COPD, severe exacerbations of COPD, DCSI score, and medications are listed in Table 1.

### The outcome of hospitalized COPD

The IRs of hospitalization for COPD were 21.5 and 20.5 per 1,000 person-years for metformin users and nonusers, respectively; the aHR was 1.34 (95% CI, 1.26–1.43), with a *p* value < 0.0001. The cumulative incidence of hospitalization for COPD (Fig. 2B) revealed a higher risk of pneumonia in metformin users relative to nonusers. The aHRs for hospitalization with COPD for < 15, 15–29, and ≥ 30 cumulative DDDs were 1.29, 1.31, and 1.42, respectively (Fig. 3A); the aHRs of the prescribed daily doses of metformin, < 500, 500–749, and ≥ 750 mg/day, were 1.20, 1.33, and 1.69, respectively (Fig. 3B).

### The outcomes of invasive and noninvasive ventilation

The IRs of noninvasive positive pressure ventilation (NIPPV) were 7.03 and 6.48 per 1,000 person-years for metformin users and nonusers, respectively; aHR was 0.99, with a 95% CI of 0.89–1.10 and *p* value of 0.95 (Table 2). The IRs of IMV were 22.2 and 18.4 per 1,000 person-years for metformin users and nonusers, respectively; aHR was 1.10, with a 95% CI of 1.03–1.17 and *p* value of 0.001. The cumulative incidence of invasive mechanical ventilation (IMV, Fig. 2C) revealed that metformin users had a higher risk of IMV relative to nonusers. With respect to the risk of IMV, the aHRs of the cumulative DDDs of metformin use of < 15, 15–29, and ≥ 30 were 1.10, 1.07, and 1.13, respectively (Fig. 3A); the aHRs of the prescribed daily doses of < 500, 500–749, and ≥ 750 mg/day of metformin were 0.90, 1.12, and 0.96, respectively (Fig. 3B).

### The outcome of lung cancer

The IRs of lung cancer were 3.6 and 3.0 per 1,000 person-years for metformin users and nonusers, respectively; the aHR was 1.12 (95% CI of 0.96–1.30), with *p* = 0.12 (Table 2).

### Sensitivity test

We conducted a sensitivity test by defining metformin use ≧ 180 days within 365 days after the new date as metformin users. Marginal structural Cox model was used to compare the outcomes of metformin users vesus matched non-users (Table 3). The metformin users were associated with higher risks of NIPPV [aHR 1.22 (1.20–1.25)], IMV [aHR 1.25 (1.22–1.27)], bacterial pneumonia [aHR 1.25 (1.22–1.28)], hospitalization for COPD [aHR 1.24 (1.21–1.26)], and lung cancer [aHR 1.22 (1.19–1.24)], as compared with non-users.

**Table 3:** Incidence rates and hazard ratios of main respiratory outcomes of metformin use ≧ 180 days vs matched non-users.

	Metformin non-users			Metformin users			Crude		Adjusted	
	Events	PY	IR	Events	PY	IR	HR (95% CI)	*p* value	HR (95% CI)	*p* value
NIPPV	693	117,055	5.92	611	122,105	5.00	0.95 (0.94–0.97)	< 0.0001	1.22 (1.20–1.25)	< 0.0001
IMV	1954	115,679	16.8	1749	120,643	14.4	0.96 (0.94–0.98)	< 0.0001	1.25 (1.22–1.27)	< 0.0001
Bacterial pneumonia	3,571	110,527	32.3	3,259	114,522	28.4	0.98 (0.96–0.99)	0.02	1.25 (1.22–1.28)	< 0.0001
Hospitalization for COPD	1847	117,943	15.6	2084	113,235	18.4	0.96 (0.95–0.98)	0.0001	1.24 (1.21–1.26)	< 0.0001
Lung cancer	368	117,429	3.1	406	122,396	3.3	0.95 (0.94–0.97)	< 0.0001	1.22 (1.19–1.24)	< 0.0001

PY, person-years; IR, incidence rate per 1,000 person-years; HR, hazard ratio; CI, confidence interval. Models adjusted by gender, age, Charlson Comorbidity Index, moderate exacerbations of COPD, severe exacerbations of COPD, DCSI score, and medications are listed in Table 1.

## Discussion

Our study observed that metformin use in patients with T2DM and COPD increased the risks of bacterial pneumonia, hospitalization for COPD and use of IMV. The sensitivity test of metformin use for more than 6 months versus control also disclosed consistent results. The relative risks of the cumulative and prescribed daily dose of metformin seemed to have dose–response relationship.

Patients with T2DM have an increased risk of respiratory infections which could lead to frequent exacerbations of COPD and worse outcomes^20^. Treatment with corticosteroids is also associated with a higher risk of pneumonia in patients with COPD^21^. An animal study suggested that the use of metformin could attenuate bacterial growth by reducing the glucose permeability of the airway^15^. However, our study observed that the use of metformin increased the risk of bacterial pneumonia. In this study, metformin use increased the risk of hospitalization for COPD, this severe exacerbation might increase the use of systemic steroids and add the hazard of bacterial pneumonia.

T2DM has been associated with progression and worse prognosis of COPD^9, 17^. Greater COPD exacerbation may result in greater airflow limitation, respiratory failure, or hospitalization. Three cohort studies have demonstrated that metformin use could reduce the risk of mortality^16–18^. Bishwakarma et al.^22^ demonstrated that metformin use in patients with coexistent COPD and T2DM was associated with a lower risk of COPD-specific emergency room (ER) visits and hospitalizations, especially in patients with low-complexity COPD. In another study using randomized control of metformin use in nondiabetic patients with severe exacerbation of COPD, metformin use had no detectable effect on clinical outcomes or C-reactive protein^19^. Our study demonstrated that metformin users were associated with higher risk of the cumulative incidence of hospitalization for COPD. The Cox proportional hazards with a marginal model indicated that relative to nonuse, metformin use had an aHR of 1.34 (95% CI 1.26–1.43). The cumulative and prescribed daily doses of metformin also had dose–response trends with respect to the risk of hospitalization for COPD. The inconsistency among these 3 studies may be attributed to differences in sample sizes, patient ethnicities, and follow-up periods. Patients with acute exacerbated COPD often have low serum vitamin B12 levels^23^. Long-term metformin use was associated with lower serum vitamin B12 levels^24^, which may affect respiratory muscle function^25^, and have relationship with the exacerbation and hospitalization for COPD.

Oxygen therapy improves survival in patients with severe lung disease and hypoxemia. Mechanical ventilation is essential life-support for respiratory distress; the slow and sustained flow of air to distal airspaces minimizes airflow turbulence, reduces airway resistance, lowers the effort required for breathing, and relieve hypercapnic respiratory failure^26^. Sexton et al.^13^ conducted a prospective observational study on 6-month metformin use in 17 participants with moderate to severe COPD and observed that metformin use was associated with reduced symptoms of dyspnea and improved health status. By contrast, our results indicated that metformin use increases the risk of IMV. This inconsistency is probably due to differences in research designs and sample sizes. Mitochondria account for most of the body’s oxygen consumption in their production of adenosine triphosphate (ATP). Metformin can enter cells and inhibit complex I of the mitochondrial electron transport chain through organic cation transporter 1. This inhibition potentially reduces ATP production, which may lead to energy stress and potential mitochondrial dysfunction^27^. Metformin can diminish mitochondrial respiration in skeletal muscle^28^. Insufficient energy in respiratory muscle may affect pulmonary function, and the degree of mitochondrial dysfunction in the skeletal muscle was associated with the severity of diseases^29^. In addition, our study exhibited that metformin use increased the risks of bacterial pneumonia and hospitalization for COPD, both of which are prone to developing into hypercapnic respiratory failure and need the support of invasive mechanical ventilation.

Metformin has been reported have anticancer effects on many types of tumor. A meta-analysis of eight observational studies by Zhu et al.^30^ suggested that metformin use can yield a significant 16% reduction in the risk of lung cancer. However, in another meta-analysis of 11 cohort and four case–control studies, Nie et al.^31^ reported a null association between metformin use and lung cancer. Likewise, our study of metformin use in patients with T2DM and COPD revealed no significant association with lung cancer.

This study has some limitations. First, the national health insurance research database (NHIRD) does not provide data regarding family medical history, social economic status, education, body weight, smoking habits, alcohol-drinking habits, or physical activity, all of which may influence our investigated outcomes. Second, because NHIRD claims data do not include symptoms, signs, or the results of pulmonary functional tests, we could not calculate COPD severity scores. Thus, to balance the severity of COPD between metformin users and nonusers, we used clinical records (antibiotics use, oral corticosteroid use, hospitalization or ER visit) to assess the number of moderate and severe exacerbations of COPD. Third, much evidence favors positive roles for long-acting β2 agonists (LABAs) and long-acting muscarinic antagonists (LAMAs) in reducing COPD exacerbations; most of our patients had moderate or severe exacerbations. However, inhaled LABA and LAMA use was not prevalent, which indicated that the treatment was suboptimal. We must educate our physicians, encourage them to adhere to the guideline of COPD treatment, to improve patient’s care. Finally, indication bias might exist in the cohort study because patients and physicians may unconsciously select their preferred method of treatment. Therefore, we balanced multiple variables, such as basic demographics, comorbidity, severity of COPD and T2DM, and the use of various medications to minimize such bias.

In conclusion, our study observed that metformin use in patients with T2DM and COPD was associated with higher risks of bacterial pneumonia, hospitalization for COPD and use of IMV. However, because of some unmeasured or inevitable bias still exist in this cohort study, stringent prospective studies or randomized control clinical trials are warranted to verify our results.

## Methods

### Study design and participants

The NHIRD comprises health care data from approximately 99% of the population of Taiwan (approximately 23 million people)^32^. The NHIRD has encrypted data on date of birth; gender; area of residence; disease coding according to the International Classification of Diseases, Ninth Revision, Clinical Modification (ICD-9-CM); prescriptions; and clinical procedures. The LHDB is part of the NHIRD. The LHDB selected 120,000 newly diagnosed diabetes patients yearly from 1999 to 2012, and their medical records from 1996 to 2013 were collected.

We involved patients who had a record of T2DM and COPD in the LHDB between January 1, 2000 and December 31, 2012. Patients’ study endpoints were withdrawal from the National Health Insurance program, occurrence of the outcome of interest, or December 31, 2013, whichever was earliest. The number of diagnoses of COPD (ICD-9-CM codes: 491, 492, and 496) was ≥ 2 for outpatients within 1 year or ≥ 1 diagnosis for hospitalization or ER visit. The criteria for the definition of COPD that use the ICD-9-CM in the NHIRD was validated in a previous study^33^. Moderate COPD exacerbation was indicated by prescription of antibiotics and/or oral corticosteroid; severe COPD exacerbation was indicated by hospitalization or an ER visit^34^. Patients were excluded if they were not between 40 and 100 years old, withdrew from the National Health Insurance program, underwent dialysis (V56.0, V56.8, V45.1), or were diagnosed with type 1 DM (250.1x), hepatic failure (570, 572.2. 572.4, 572.8), or lung cancer (162) before the index date. We also excluded patients who had been diagnosed with T2DM or COPD before January 1, 2000. We confirmed that all methods were performed in accordance to Declaration of Helsinki. This study was approved by the Institutional Review Board of China Medical University in central Taiwan (CMUH104-REC2-115). All information that could be used to identify patients or care providers was encrypted before release to protect participants’ privacy. Therefore, we were granted to waive of the informed consent.

### Procedures

**Figure 4:**
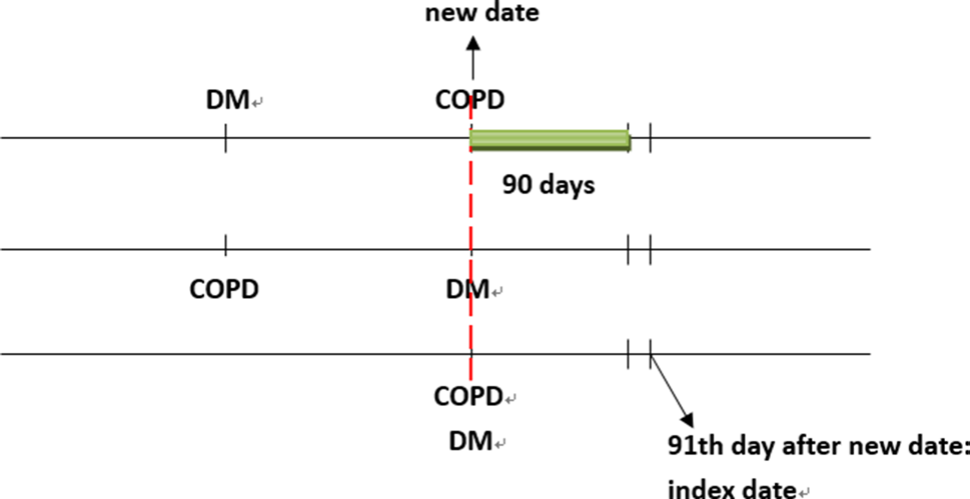
We defined patients who have ever taken metformin within 90 days after new date as metformin users; the 91th day after new date as the index date.

The date of concurrent diagnosis of T2DM and COPD was considered the new date. Within 90 days after the new date, patients who took metformin for at least 30 days were considered metformin users. Those who had not used metformin were considered metformin nonusers. The 91st date after the new date was considered the index date (Fig. 4). We identified potential explanatory variables, such as baseline characteristics, moderate or severe exacerbation of COPD, history of bacterial pneumonia, Charlson Comorbidity Index (CCI)^35^, Diabetes Complications Severity Index (DCSI) score^36^, and the use of specific classes of drugs (such as antidiabetics other than metformin, antihypertensive drugs, COPD drugs, statins, and aspirin). CCI and DCSI scores were calculated using patients’ status within 1 year before the index date.

### Main outcomes

The main outcomes were hospitalized bacterial pneumonia (481, 486, 482.41, and 482.8), hospitalization for COPD, the use of noninvasive positive pressure ventilation (NIPPV, 93.90 and 93.91) or invasive mechanical ventilation (IMV, 96.7), and lung cancer. To preclude insufficient exposure (metformin use) time, patients who were admitted for bacterial pneumonia or COPD, used NIPPV or IMV, developed lung cancer, or died within 180 days after the index date were excluded. We have performed a sensitive test by defining the index date at 366th day after the new date, defining patients who used metformin ≧ 180 days within the 365 days between the new date and index date as metformin users, to compare the respiratory outcomes between the matched metformin users and control.

### Statistical analyses

To maximize comparability, a propensity score matching was used to balance user and nonuser groups with respect to known variables^37^. We determined propensity scores through nonparsimonious multivariable logistic regression, with receipt of metformin as the dependent variable. We incorporated 35 clinically relevant covariates into our analysis as independent variables (Table 1). We applied the nearest-neighbors algorithm to match pairs, assuming that proportions of 0.995–1.0 were acceptable.

For the main outcomes, we censored patients at either the time of events or end of the follow-up on December 31, 2013, whichever came earlier. Using the Fine and Gray’s sub-distribution hazard model, we compared the cumulative incidence of events over time between metformin users and nonusers. We used a marginal structural Cox model, and robust sandwich standard error estimates to compare the outcomes while controlling for baseline covariates^38^. All analyses were done on the as-treated basis. We stopped following the metformin users when they discontinued metformin after the index date; on the contrary, the metformin non-users were censored when they started to use metformin after the index date. To assess dose effect, we analyzed the relative risks of bacterial pneumonia, hospitalization for COPD and IMV with regard to the cumulative DDD of metformin during the 90-day observational period (< 15, 15–29, and ≥ 30 DDD) and prescribed daily dose (< 500 mg, 500–749 mg, and ≥ 750 mg). DDD is a unit of measurement defined as the assumed average maintenance dose per day as the main indicated drug in an adult; the DDD is 2,000 mg for metformin. We report results as hazard ratios with 95% CI. A two-tailed *p* value less than 0.05 indicates significance. We used SAS statistical software (Version 9.4 for Windows; SAS Institute, Inc., Cary, NC, USA) for data analysis.
